# Retinal Vessel Diameter Measurement Using Unsupervised Linear Discriminant Analysis

**DOI:** 10.5402/2012/151369

**Published:** 2012-11-06

**Authors:** Dinesh K. Kumar, Behzad Aliahmad, Hao Hao

**Affiliations:** School of Electrical and Computer Engineering, RMIT University, 124 Latrobe Street, Melbourne, VIC 3000, Australia

## Abstract

An automatic vessel diameter measurement technique based on linear discriminant analysis (LDA) has been proposed. After estimating the vessel wall, the vessel cross-section profile is divided into three regions: two corresponding to the background and one to the vessel. The algorithm was tested on more than 5000 cross-sections of retinal vessels from the REVIEW dataset through comparative study with the state-of-the-art techniques. Cross-correlation analyses were performed to determine the degree to which the proposed technique was close to the ground truth. The results indicate that proposed algorithm consistently performed better than most of other techniques and was highly correlated with the manual measurement as the reference diameter. The proposed method does not require any supervision and is suitable for automatic analysis.

## 1. Introduction

 Retina images allow noninvasive viewing of the in-vivo vessels and have been established as indicator for incidence of diabetic retinopathy [1, 2], early indicator of stroke [3, 4] and hypertension [5]. It is the best modality to see the microvascular abnormalities [6] such as change in the width of the vasculature. Changes in the width of the retinal arteriole and venules are known as direct indictors of retinal vasculature abnormality [7]; detection of which requires accurate measurement of retinal vessel diameter. However, complex background and uneven lighting conditions result in poor contrast at vessel edges [8], and this result in inaccurate diameter measurement.

 Several techniques have been published previously for vessel diameter estimation and edge delineation. Brinchmann-Hansen and Heier proposed the Half Height Full Width (HHFW) method in which the diameter was defined as the distance between the points on the vessel intensity cross-section profile where the function reaches 50% of its maximum value to either side of the estimated centre point [9]. Gregson et al. [10] fitted a rectangle to the vessel profile and estimated the width by setting the area under the curve equal to the area under the rectangle. In [11], the vessel profile was approximated by 1D Gaussian function based on the assumption that the intensity profile follows a symmetric Gaussian-like shape. This was further extended to 2D Gaussian by Lowell et al. [12] which was more robust compared to 1D Gaussian method. Gao et al. [13] established that the retinal vessel profile could be fitted with twin-Gaussian model. The study found a linear relationship between the standard deviation (SD) of the Gaussian and the gold standard diameters obtained from the sharper images of the angiograms. However, these methods may fail when the fitted curves do not converge to the model. An analysis was performed by Chapman et al. [14] to compare different automated vessel diameter measurement algorithms, showing that the sliding linear regression filter (SLRF) was more precise than the twin-Gaussian technique. However, the SLRF method relied on a parameter from the twin-Gaussian analysis to adjust its window size. Al-Diri et al. [15] proposed the extraction of segment profiles (ESP) algorithms based on growing a Ribbon of Twins (ROT) and active contour model over segmented vessels. Two merging pairs of contours were used at each edge (one inside and one outside the vessel) to converge to the boundary and delineate the edges for diameter measurement. The importance of independent boundary extraction led to the work by Xu et al. [8]. They established a graph-based method in which the vessel boundaries were segmented simultaneously using a 3D surface segmentation scheme. While a number of these techniques have been shown to be accurate [8, 15], asymmetry of the vessels' cross-section profile due to uneven illumination [11], limitations of the imaging equipment, and blurred vessel boundaries can result into incorrect detection of real edge location and therefore imprecise diameter estimation by different algorithms and observers [8].

 In order to overcome the above limitation, a new vessel diameter measurement based on an Unsupervised Linear Discriminant Analysis (ULDA) [16] has been proposed. The technique does not require any supervised training and is suitable for automation purpose. It was assessed on the publically available REVIEW [17] dataset and compared with sate of the art methodology techniques. 

## 2. Unsupervised Linear Discriminant Analysis Diameter Measurement (ULDM)

We have proposed, developed, and tested reliability of ULDM to automatically measure the diameter of the retinal vasculature. ULDM does not require any supervision, and the grader only has to identify the region of interest (ROI). After the ROI has been identified by the grader, the vessel boundaries are estimated, and the intensity cross-section profiles are obtained during the initialisation step. The next step is to automatically generate the training data using the Linear Discriminant Analysis (LDA) classifier [18] which is trained to separate the profile into the three Sections; 1 corresponding to the vessel surface and the Sections 2 and 3 corresponding to the background at either side of the vessel. The trained LDA identifies the three sections, and the width of Section 1 is taken as the vessel diameter. These steps are explained below in Section 2.1, and LDA has been explained in Sections 2.2 and 2.3. 

### 2.1. Initialization

 During the initialization phase, the vessel boundaries are automatically estimated and tracked using the vector sum of image Hessian eigenvectors on circles centered at vessel edges [19]. Using this method, the pixels corresponding to the vessel boundaries are identified. 

 After estimating the vessel boundary, the intensity profile along a line normal to the vessel edges and greater than the actual vessel diameter is obtained for vessels with diameter greater than 3 pixels. The length of the normal line was set to 10% greater than the shortest Euclidean distance between the estimated edge points to cover the background intensities corresponding to its either side while not overlapping the intensities for adjacent vessels. The normal line corresponds to the shortest Euclidean distance between the points on vessel boundaries. However, for fine vessels with distance between the two boundaries equal to or less than 1 pixel the normal cannot be estimated from the edges. In such a case, the normal is estimated from the direction of the progress of the vessel tracking as in [20]. This initialization process estimates the boundary of the vessels and is used only for cross-section profile recording. However, this is not suitable for measuring the diameter as tracking methods are sensitive to illumination conditions and not suitable for subpixel measurement accuracy [21]. 

### 2.2. Training LDA

 Linear discriminant analysis is a method for data classification and dimensionality reduction [18]. LDA optimizes class separability by maximizing the ratio of interclass to intraclass variances and is suitable for applications with unequal sample sizes. This was applied to the retinal vessel cross-section profiles after the average intensity value was subtracted and was trained to classify the intensities into the three sections (i) vessel surface, (ii) background to the left, and (iii) background to the right of the vessel to detect the points discriminating vessel from its background. 

 The LDA is a supervised technique, and the training process has to be done automatically so that the system is suitable for unsupervised and automatic analysis. This is performed using a cluster analysis technique, based on the sharp transitions on vessel profile corresponding to the edges [11–13]. However, uneven illuminations, background noise, central light reflex phenomena [9], overlying structures of the eye, and the capturing equipment [9, 19] distort the profile of the vessel. Profile distortion is defined as any change in the axial symmetry, shape, and smoothness compared to the inverse single Gaussian function as the model of retinal vessel profile [11]. As in our case, deliberate inclusion of some pixel intensities corresponding to the background region into the vessel profile highlights the distortions as a number of extrema points on the profile. These points which mainly appear in the area corresponding to the background region were used for cluster analysis to obtain the LDA training classes. 

**Figure 1:**
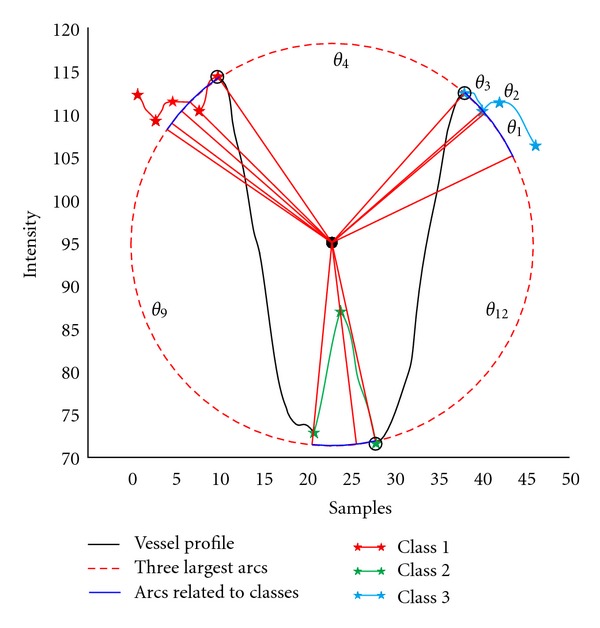
Obtaining the training classes for a sample vessel profile (CLRIS, image no. 1, cross section no. 22). The extremums related to the three classes are shown with asterisks in different colours (Red, Green, and Blue). Blue curves are the regions related to the detected classes. Red curves are the boundaries separating the classes. *θ*_4_, *θ*_9_, and *θ*_12_ represent the first three largest angles.

In order to perform cluster analysis to identify the extrema points, the differential of the profile is computed. In the differential of the profile plot, the zero-crossing points corresponding to the location of the extrema are obtained to find three critical points as the reference to initiate cluster analysis. According to the Gaussian model of vessel profile [11, 12], it is confirmed that generally the edge points have high intensity values while the points on the surface of the vessels contain lower intensities except for the central light reflex area. Therefore, location of the lowest minima in intensity which correspond to the vessel region is marked as the first required critical point. The two highest maxima at both side of this point are labelled as the two other points each correspond to one background region at left and right side of the vessel. Given the three points guarantees the existence of a circle to pass through them by joining the vertices in pairs (creating chords) and forming a triangle; as the perpendicular bisectors of the chords always pass through the centre of a circle which includes those vertices. This circle is used as the basis for cluster analysis and unsupervised classification of the local extremums to train the LDA. An example of a vessel cross-section profile with centre light reflex (CLR) is shown in Figure 1. As shown in this figure, from the centre of this circle a radial line was connected to each point. Starting from 0 degree and rotating anticlockwise, all the angles (*θ*_1_, *θ*_2_,…, *θ*_*n*_) between the two consecutive radial lines were obtained sequentially and sorted from the largest to the smallest value. The first three largest angles corresponding to the largest arcs on the circle were considered as the boundaries separating the three training classes (e.g., *θ*_4_, *θ*_9_, and *θ*_12_ in Figure 1). All the extrema that fell within the same section between the two boundary arcs were categorized as the same class. Group 1 and 3 were considered as the maxima and minima relating to the background at either side of the vessel and group 2 to the extremums corresponding to the trunk as shown by asterisks in a unique colour (Figure 1). In order to increase the number of training samples for more accurate cluster analysis, all the points on the vessel profile within the distance between the two horizontally farthest extremums of each class were considered as the member of that class and used as training samples.

### 2.3. Linear Discriminant Analysis (LDA)

 Linear discriminant analysis has gained popularity in feature extraction and pattern recognition due to its uncomplicated processing as well as providing higher discrimination power compared to the principal component analysis (PCA) as another alternative method for data classification and dimensionality reduction [18]. Therefore, a generalization of the Fisher's LDA (1936) is applied to the intensities of retinal vessel cross-section profiles after the mean value is removed, in order to classify them into three major regions based on their physical location on the profile (vessel or background). The decision boundaries are used to find the midpoint between the maximum and minimum intensities corresponding to the vessel edges and to measure the diameter based on the definition available in Gang et al. [22]. The obtained training samples (intensities versus sample) with three known classes from Section 2.2 are given as the training inputs to estimate the corresponding dispersion matrices according to [16], train the classifier, and obtain three discriminant functions (DFs) corresponding to the three classes. This method is valid due to the robustness of linear discriminant function (LDF) to unequal dispersion matrices and the assumption of similarity existence between the three sample dispersion matrices [16]. 

### 2.4. Diameter Measurement

**Figure 2:**
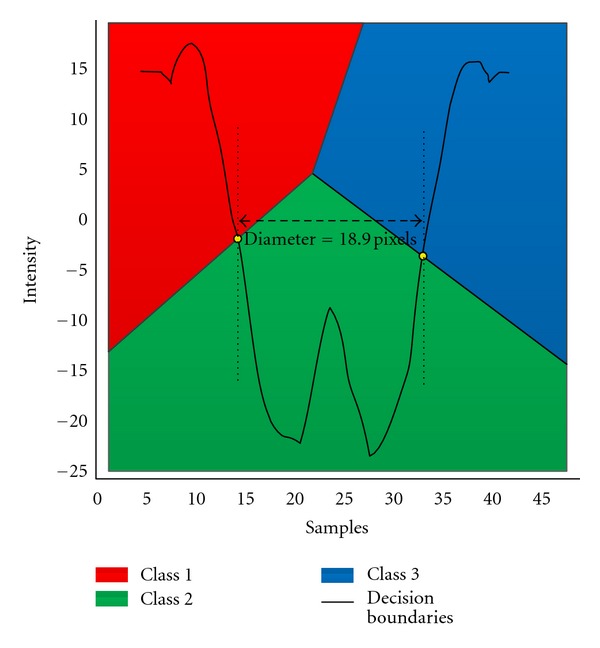
Example of ULDM output showing a vessel cross-section profile (CLRIS, image no. 1, cross-section no. 22) with classified padded intensity values and the decision boundaries between the classes. The horizontal distance between the two points where the decision boundaries cross the vessel edges (approximately at 50% intensity change) was considered as the diameter (18.9 pixels in this example).

In order to measure the diameter, the intensity versus sample plane of the vessel profile is padded with a set of test points as input to the classifier. Given these points, the trained LDA generates three decision boundaries corresponding to the three training classes. Two of these intersect with the intensity profile and segment the profile into three regions. The intersection points with the vessel profile specify the midpoint between the maximum and minimum intensities corresponding to the vessel edges. The horizontal distance between the two midpoints gives the vessel diameter (Figure 2).

## 3. Materials

 The publicly available REVIEW database was used to assess the ULDM vessel diameter measurement technique. This is a widely accepted database of retinal images and has four datasets of mixed quality images [8, 17]. It contains 5066 vessel diameters measured by three different observers from 193 segments. Only the green channel was used as it provides better contrast between the vessels and the background [23]. The database is summarized as follows.

1. Kick-Point Image Set (KPIS) dataset, consisting of two good quality retina images (288 × 119 and 170 × 192 pixels) with 3 segments and 164 cross-sectional measurements. The edges were determined based on the kick-points present in cross-sections of the vessels. The kick-points are normally observed in highly focused retina images with sharp transition from the background intensity to the vessel edges.
2. High Resolution Image Set (HRIS) represents different severity of diabetic retinopathy. The abnormalities that appear near the vessel edges provide a challenge when trying to determine the vessel diameter. It contains four images (3584 × 2438 pixels each) and 90 segments with 2368 manually marked profiles.
3. Central Light Reflex Image Set (CLRIS) represents exaggerated vascular light reflex which appears as a small Gaussian in the middle of the vessel profile. CLRIS consists of two retinal images (2160 × 1440 pixels each) with total number of 21 segments and 285 cross-sections.
4. Vascular Disease Image Set (VDIS) includes eight noisy retina images (1360 × 1024 pixels each), six of which suffer from diabetic retinopathy. It consists of 79 segments and 2249 measured profiles. The VDIS dataset provides a greater challenge to the diameter measurement due to its inherent variations.

## 4. Data Analysis

ULDM was compared with the HHFW, 1D and 2D Gaussian models, ESP, and the graph based on comparison method as provided in [8]. The edge points of more than 5000 cross-sections from the REVIEW database were used as ground truth to validate the technique. Following the result evaluation and presentation method used in [8] the average, *μ*_1_, and standard deviation (SD), *σ*_1_, of the estimated diameters were calculated in pixels for each single database and presented together with the reported values from other methodologies. The signed *μ*_2_ and corresponding *σ*_2_ were also defined as the average and SD of point by point difference between the measured and the reference diameter. The reference was considered as the average of the three diameter values measured by the three observers.

 For performance and efficiency estimation, the success rate was first calculated as the ratio of the number of measureable cross-sections to the total number of available profiles in the database reported by observers. The higher success rate and lower deviation from the reference diameter (lower *μ*_2_ and *σ*_2_) indicated more precise estimation. Measure of similarity between the ULDM and the three different manual measurements and the ground truth measurements (average) was obtained by cross-correlation analysis performed on all the reported segments in the database (Table 5).

## 5. Results

 Tables 1, 2, 3 , and 4 give the comparison of the accuracy and precision between the three manual measurements (observer 1, 2, and 3), the HHFW [9], 1D Gaussian [11], 2D Gaussian [12], ESP [15], graph-based [8], and the proposed ULDM techniques. The four tables compare the four different datasets of the REVIEW database. The Tables 1 to 4 correspond to the HRIS, CLRIS, VDIS, and KPIS datasets, respectively. The reporting format in these tables is the same as the one used by previous studies [8, 15], with the addition of row corresponding to the proposed technique, ULDM. 

 The second column of these four tables presents the success rate in percent corresponding to each measurement method as defined earlier. From this column of Tables 1–4, it is observed that ULDM has a high success rate similar to other five computerized diameter measurement techniques; HHFW, 1D Gaussian, 2D Gaussian, ESP, and graph based. The Average value of the estimated vessel widths *μ*_1_ together with the standard deviation *σ*_1_ from the mean is provided in columns three and four, respectively. Columns 5 and 6 are the indicator of the signed average (*μ*_2_) and SD (*σ*_2_) of the difference between the measured diameter and the gold-standard diameter obtained by averaging the three manual measurements, respectively. From these columns, it is observed that different techniques have the least difference from the gold standard for the different datasets. While 2D Gaussian has the smallest *μ*_2_ for HRIS dataset, graph-based method has the smallest *μ*_2_ for CLRIS dataset, ESP has the smallest *μ*_2_ for VDIS dataset, and ULDM has the smallest *μ*_2_ for KPIS dataset. From these tables, it is observed that while other techniques are more suited for one dataset, ULDM is more consistent and has the smallest or second smallest error for all the datasets.

While the above reporting technique [8, 15] provides the measure of the accuracy in terms of average diameter difference, this does not provide a good measure of precision to compare the proposed method with other methodologies or the manual measurements. Therefore cross-correlation analysis was performed for further investigation and is reported in Table 5. In Table 5, the columns 2, 3, and 4 correspond to the correlation between the three manual measurements, the forth is of the average of the three manual measurements that results in Obs_avg, while the column 6 is that of the ULDM. This was repeated for each of the four datasets. The correlation matrix being symmetrical, only the lower half is reported. As expected, the diagonal elements are unity. From this table it is observed that the correlation between ULDM and the Obs_avg (bench mark) is similar to the correlation between the three manual measurements and Obs_avg. For HRIS, VDIS, and CLRIS datasets, this ranged between 0.87 and 0.91, while for the KPIS, this was significantly lower (0.52). 

## 6. Discussion

 In this work, an unsupervised retinal vessel diameter measurement technique has been proposed and validated using expert annotated publically available dataset (REVIEW database) through comparison with other state-of-the-art methodologies. The advantage of this method is that it does not require any supervision and measures the vessel diameter to subpixel accuracy. The results also show that while other techniques are biased towards a specific dataset, this method appears to have a good accuracy for all the datasets. 

 According to Tables 1 to 4, among the four datasets, the proposed method had the highest performance on the KPIS with 100% success rate and the signed mean and SD difference of 0.50 and 0.60, respectively. The test on HRIS resulted into the success rate of 99.6% and the signed mean and SD difference of 0.21 and 0.79. This success rate degradation was mainly due to the appearance of diabetic abnormalities near the vessel boundaries which is still a challenge among all measurement techniques. The central light reflex validation test on CLRIS dataset revealed the success rate of 98.2% with −0.55 and 1.79 as the mean and SD difference. The proposed technique indicated the success rate of 96.3% when tested on the noisy pathological images (VDIS dataset) with mean and SD difference of −0.64 and 1.18, respectively.

As stated before, the earlier reporting method does not determine the precision of the measurements. For this purpose, the correlation between the ULDM, the manual measurements, and the Obs_avg as the benchmark has also been reported (Table 5). This reporting is essential to establish the precision of the measurements by providing point by point similarity comparison between the measured diameters and the ground truth. According to the cross-correlation analysis in Table 5, the ULDM and Obs_avg were highly correlated with each other when tested on HRIS, VDIS, and CLRIS databases with the correlation coefficients of 0.87, 0.90, and 0.91, respectively. The lowest value of 0.52 was obtained for the KPIS database. This was expected as the interobserver correlation of 0.6 denoted measurement inconsistencies and poor correlation even between the three experts' estimations making the KPIS very challenging image set to the graders.

Overall, the ULDM was found to be a robust technique for retinal vessel diameter measurement against the images representing different severity of diabetic retinopathy, vascular disease, and exaggerated vascular light reflex. The values measured by the proposed technique showed very similar trend to average of the ones reported by the observers. In future, it is expected to have potential applications in medical section for quantification and early detection of retinal and cardiovascular diseases.

## Figures and Tables

**Table 1:** Comparison of vessel diameter measurement accuracy and precision between the proposed ULDM, established techniques, and manual measurement for the HRIS database^a^.

HRIS database					
Method name	Success rate (%)	Diameter		Difference	
		Average *μ*_1_	SD *σ*_1_	Average *μ*_2_	SD *σ*_2_
Observer 1	100	4.12	1.25	−0.23	0.288
Observer 2	100	4.35	1.35	0.002	0.256
Observer 3	100	4.58	1.26	0.23	0.285
HHFW	88.3	4.97	—	0.62	0.926
1D Gaussian	99.6	3.81	—	−0.54	4.137
2D Gaussian	98.9	4.18	—	−0.17	6.019
ESP method	99.7	4.63	—	0.28	0.42
Graph-based method	100	4.56	1.30	0.21	0.567
Proposed ULDM	99.6	4.19	1.35	0.21	0.79

**Table 2:** Comparison of vessel diameter measurement accuracy and precision between the proposed ULDM, established techniques, and manual measurement for the CLRIS database^a^.

CLRIS database					
Method name	Success rate (%)	Diameter		Difference	
		Average *μ*_1_	SD *σ*_1_	Average *μ*_2_	SD *σ*_2_
Observer 1	100	13.19	4.01	−0.61	0.566
Observer 2	100	13.69	4.22	−0.11	0.698
Observer 3	100	14.52	4.26	0.72	0.566
HHFW	0	—	—	—	—
1D Gaussian	98.6	6.3	—	−7.5	4.137
2D Gaussian	26.27	7.0	—	−6.8	6.019
ESP method	93.0	15.7	—	−1.90	1.469
Graph-based method	94.1	14.05	4.47	0.08	1.78
Proposed ULDM	98.2	13.23	3.55	−0.55	1.79

**Table 3:** Comparison of vessel diameter measurement accuracy and precision between the proposed ULDM, established techniques, and manual measurement for the VDIS database^a^.

VDIS database					
Method name	Success rate (%)	Diameter		Difference	
		Average *μ*_1_	SD *σ*_1_	Average *μ*_2_	SD *σ*_2_
Observer 1	100	8.50	2.54	−0.35	0.543
Observer 2	100	8.91	2.69	0.06	0.621
Observer 3	100	9.15	2.67	0.30	0.669
HHFW	78.4	7.94	—	−0.91	0.879
1D Gaussian	99.9	5.78	—	−3.07	2.110
2D Gaussian	77.2	6.59	—	−2.26	1.328
ESP method	99.6	8.80	—	−0.05	0.766
Graph-based method	96.0	8.35	3.00	−0.53	1.43
Proposed ULDM	96.3	8.68	2.82	−0.64	1.18

**Table 4:** Comparison of vessel diameter measurement accuracy and precision between the proposed ULDM, established techniques, and manual measurement for the KPIS database^a^.

KPIS database					
Method name	Success rate (%)	Diameter		Difference	
		Average *μ*_1_	SD *σ*_1_	Average *μ*_2_	SD *σ*_2_
Observer 1	100	7.97	0.47	0.45	0.234
Observer 2	100	7.60	0.42	0.08	0.213
Observer 3	100	7.00	0.52	−0.52	0.233
HHFW	96.3	6.47	—	−1.05	0.389
1D Gaussian	100	4.95	—	−2.57	0.399
2D Gaussian	100	5.87	—	−1.65	0.337
ESP method	100	6.56	—	−0.96	0.328
Graph-based method	99.4	6.38	0.59	−1.14	0.67
Proposed ULDM	100	7.02	0.67	−0.50	0.60

**Table 5:** Cross-correlation comparison between the ULDM, manual measurements, and their average value.

		Observer 1	Observer 2	Observer 3	Obs_avg^a^	ULDM
HRIS	Observer 1	1				
	Observer 2	0.93	1			
	Observer 3	0.91	0.94	1		
	Obs_avg	0.97	0.98	0.97	1	
	ULDM	0.83	0.86	0.87	0.87	1

CLRIS	Observer 1	1				
	Observer 2	0.96	1			
	Observer 3	0.97	0.96	1		
	Obs_avg	0.99	0.98	0.99	1	
	ULDM	0.89	0.88	0.89	0.90	1

VDIS	Observer 1	1				
	Observer 2	0.93	1			
	Observer 3	0.92	0.90	1		
	Obs_avg	0.97	0.97	0.96	1	
	ULDM	0.88	0.90	0.87	0.91	1

KPIS	Observer 1	1				
	Observer 2	0.69	1			
	Observer 3	0.65	0.64	1		
	Obs_avg	0.90	0.87	0.87	1	
	ULDM	0.50	0.47	0.41	0.52	1

^a^Obs_avg is the data sequence containing average of vessel diameters measured by the three observers for each vessel cross-section.
